# Grazing by large savanna herbivores indirectly alters ant diversity and promotes resource monopolisation

**DOI:** 10.7717/peerj.6226

**Published:** 2019-01-11

**Authors:** Jean Purdon, Catherine L. Parr, Michael J. Somers

**Affiliations:** 1 Eugène Marais Chair of Wildlife Management, Centre for Invasion Biology, Mammal Research Institute, University of Pretoria, Pretoria, South Africa; 2 School of Environmental Sciences, University of Liverpool, Liverpool, United Kingdom; 3 Environmental Change Institute, School of Geography and the Environment, University of Oxford, Oxford, United Kingdom

**Keywords:** Ants, Grazing lawns, Bunch grass, Resource monopolisation, Competitive interactions, Assemblages, Body size, Herbivory

## Abstract

In savannas, grazing is an important disturbance that modifies the grass layer structure and composition. Habitat structural complexity influences species diversity and assemblage functioning. By using a combination of natural sites and manipulated experiments, we explored how habitat structure (grazing lawns and adjacent bunch grass) affects ant diversity and foraging behaviour, specifically the efficiency of resource acquisition, resource monopolisation and ant body size. We found that in the natural sites there was no difference in the amount of time ants took to locate resources, but in the manipulated experiments, ants were faster at locating resources and were more abundant in the simple treatments than in the more complex treatments. Ant body size was only affected by the manipulated experiments, with smaller ants found in the more complex treatments. In both the grazing lawn and bunch grass habitats there were differences in assemblage patterns of ants discovering resources and those dominating them. Seasonality, which was predicted to affect the speed at which ants discovered resources and the intensity of resource monopolisation, also played a role. We show that ants in winter monopolised more baits and discovered resources at a slower rate, but only at certain times within the experiment. Grazing in conjunction with season thus had a significant effect on ant diversity and foraging behaviour, with dominant ants promoted where habitat complexity was simplified when temperatures were low. Our results indicate that structural complexity plays a major role in determining ant assemblage structure and function in African savannas.

## Introduction

Determining the factors that govern species diversity, interactions and patterns is essential to understanding ecological processes and functioning (Oliver et al., 2015). Habitat structure is one factor that has been shown to influence faunal behaviour and diversity (e.g., Hurlbert, 2004; Tews et al., 2004; Lassau & Hochuli, 2005; Lassau et al., 2005). High structural complexity may reduce predator–prey interactions as a greater number of interstices provides more refuge areas (Almany, 2004; Grabowski, 2012). This is because as structural complexity increases, so too does the number of interstices. These interstitial sizes will decrease, excluding certain species of a particular size class, restricting their foraging area and altering competitive interactions (Kaspari & Weiser, 1999; Parr, Parr & Chown, 2003; Farji-Brener, 2004; Sarty, Abbott & Lester, 2006). As the structural complexity of a habitat decreases, the size and number of the interstices decrease, enabling species to access and locate resources more efficiently. This will then increase the number of encounters among species, resulting in competition for resources (Gibb & Parr, 2010). This is where the size grain hypothesis comes into play; this hypothesis maintains that as terrestrial walking organisms get smaller, their environment becomes less planar and more rugose (Kaspari & Weiser, 1999; Parr, Parr & Chown, 2003; Farji-Brener, 2004; Sarty, Abbott & Lester, 2006). It could therefore be predicted that the more structurally complex the habitat, the smaller the size of the organisms in it. Given that habitat complexity can influence species interactions, disturbances that alter long-term vegetation structure are likely to have an indirect influence on faunal assemblages and structure. For example, in the savannas of northern Australia, ants were found to disperse seeds significantly farther after a fire that consumed much of the ground layer (Parr et al., 2007).

In savannas, grazing by large mammalian herbivores is an important disturbance that can significantly influence habitat structural complexity through changes to the grass layer structure (McNaughton, 1985; Archibald et al., 2005; Cromsigt et al., 2017). Under intense grazing, bunch grasses (e.g., *Themeda trianda, Eragrostis curvula* and *Hyparrhenia filipendula*, in southern Africa), which are less resilient to disturbances (Coughenour, 1985), are replaced by patches of short, stoloniferous grasses (e.g., *Urochloa mosambicensis, Digitaria longifolia,* and *Sporobolus nitens*)—also called grazing lawns (McNaughton, 1985; Archibald et al., 2005; Cromsigt, 2006; Cromsigt et al., 2017). In addition to grazing, fire and high nutrient availability also play important roles in the formation, modification and maintenance of grazing lawns (McNaughton, 1979; McNaughton, 1985; Painter & Belsky, 1993; Cromsigt, 2006; Cromsigt et al., 2017).

Grazing lawns are structurally simple habitats, particularly at ground level where there are large areas of bare ground. The mosaic of short grass patches within a matrix of taller bunch grass creates additional heterogeneity within savannas. This heterogeneity is thought to increase the diversity of the ecosystem and promote resilience (Hempson et al., 2015; Howison et al., 2017a). Large differences in species composition between lawn and bunch grass systems have been reported for plant, bird and spider groups (e.g., Krook, Bond & Hockey, 2007; Hempson et al., 2015). However, while a shift in grassland type can alter diversity, it is less clear what the functional responses of biodiversity are. For example, Andersen (1991) found that hot climate specialists, the dominant *Iridomyrmex* ant species, were more prevalent in annually burnt habitats. This was because burnt habitats had higher ground temperatures because of high radiation resulting from structural simplicity.

Ants (Hymenoptera: Formicidae) make an excellent study group to explore the effects of habitat complexity as they are known to be affected by habitat structure (Lassau & Hochuli, 2004; Parr et al., 2004), and they play an important functional role in ecosystems. In savanna ecosystems, ants are diverse and highly abundant. They are also important consumers acting as specialist predators, scavengers, seed dispersers and omnivores (Andersen, 1995; Sanders & Platner, 2006) and contribute significantly to nutrient cycling and soil turning (Folgarait, 1998; James, 2004). Ants have been shown to remove most food resources in tropical habitats (Griffiths et al., 2018) and therefore changes in their abundance and activity could have important implications for resource removal rates.

This study aimed to determine how grass habitat structural differences caused by large mammal grazing affects (1) the diversity and composition of ant assemblages, and (2) food resource acquisition by ants (i.e., the ability of ants to locate and capture food resources in areas of varying habitat complexity and in different seasons). We predict that because structural simplicity facilitates movement through the environment, resources in the grazing lawn sites will be discovered at a faster rate, and because it is easier to recruit nest-mates in a simple environment, more baits will be dominated than those in the bunch grass sites. As ants are thermophilic, we predict that season will also have an influence on their assemblages, with fewer ants taking longer to discover resources in winter. To address these questions, we used both a natural and manipulative experiment. Finally, we aimed to explore (3) whether ant body size changes as a function of habitat complexity, to examine the size grain hypothesis. We predict that the grazing lawn habitat will support assemblages of more, larger bodied ants.

## Methods

### Study area

The study was conducted in Hluhluwe-iMfolozi Park (HiP), run by KZN Ezemvelo Wildlife, situated in northern KwaZulu-Natal province, South Africa (between 28°00′S–28°26′S and 31°43′E–32°09′E) (Macdonald, 1983). HiP is 900 km^2^ in size with a wide variation in altitude and rainfall. For this study, sampling was restricted to lower elevations in the iMfolozi section of the park where grazing lawns are common. The majority of the rainfall occurs in the summer between October and March with a mean of 691 mm ± 30 mm SE in the lower elevated areas of iMfolozi (Howison et al., 2017b). Average daily temperatures vary between a minimum of 13 °C and a maximum of 35 °C (Cromsigt, 2006). iMfolozi is characterised by semi-arid savanna (Whateley & Porter, 1983).

**Figure 1:**
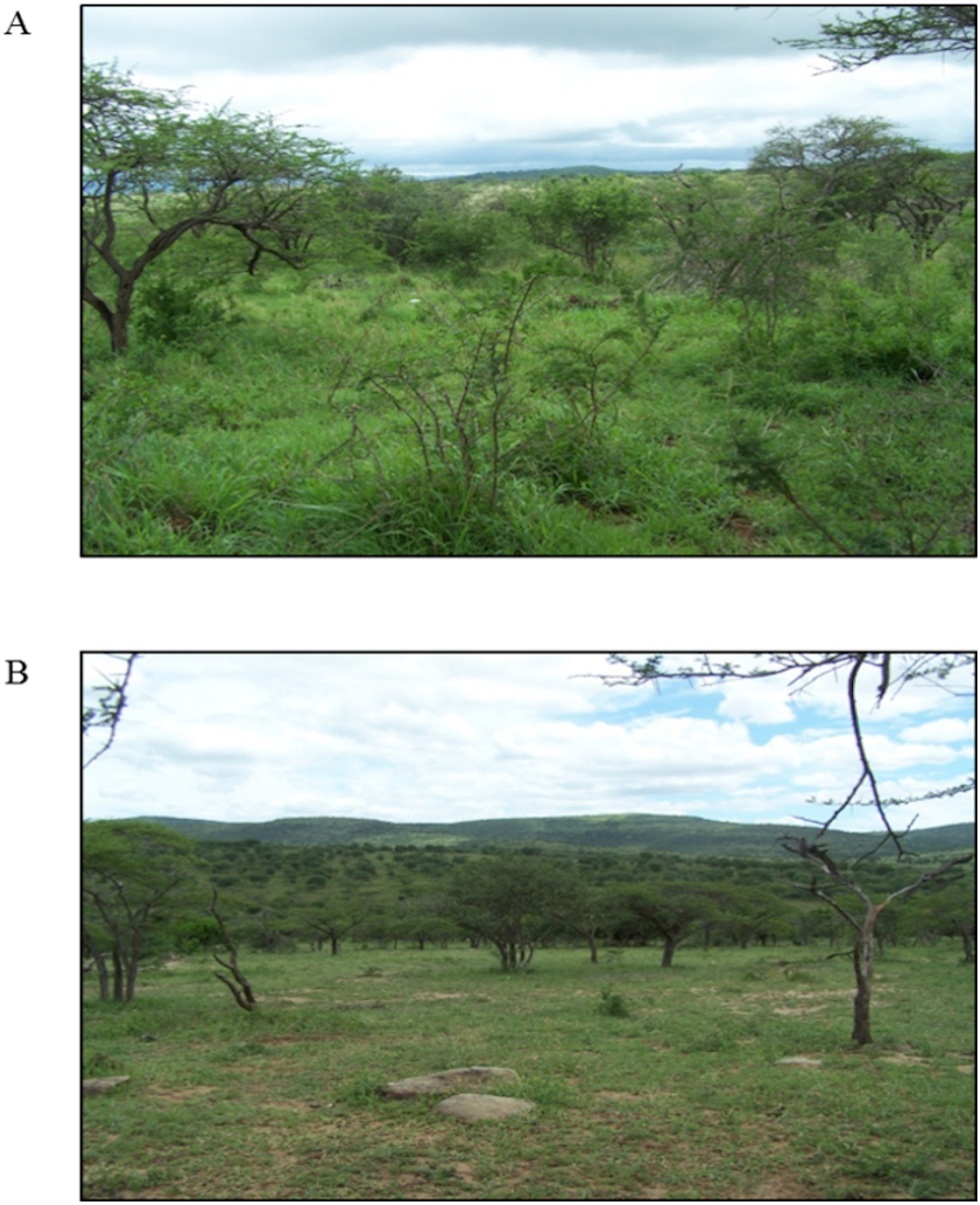
Photographs depicting (A) the complex bunch grass habitat and (B) the simple grazing lawn habitat in Hluhluwe-iMfolozi Park, South Africa.

Many of the resident species in HiP are grazing mammals (e.g., impala *Aepyceros melampus*, plains zebra *Equus quagga*, blue wildebeest *Connochaetes taurinus*), which have increased in total numbers since the area became protected and fenced in 1895. Increased mammalian herbivory has created a number of grazing lawns and these range in size from a few square meters to several hectares covering approximately 10% of the grassy areas of HiP (Archibald et al., 2005; Waldram, Bond & Stock, 2008; Cromsigt et al., 2017). Grazing lawn sites are interspersed with areas subjected to lower grazing intensity. These areas are covered with trees (e.g., *Vachellia karroo* and *Vachellia nilotica*), bush and taller bunch grasses (e.g., *Themeda triandra* and *Heteropogon contortus*) (Waldram, 2005). Sampling was carried out on six bunch grass sites (structurally complex habitat) (Fig. 1A) and six grazing lawn sites (structurally simple habitat) (Fig. 1B).

### Vegetation structural complexity and composition

We quantified microhabitat structural complexity following methods used by Gibb & Parr (2010). Measurements included vertical complexity of vegetation, presence or absence of bare ground, and height of ground vegetation. For this study, quantification of the microhabitat structural complexity in both the grazing lawn and bunch grass habitats was conducted along 80 m transects. Where a continuous transect was not possible, multiple shorter transects totalling 80 m were used. The vertical complexity of the two habitats was quantified by placing a pole vertically on the transect at 8 m intervals. The pole had four 5 × 6 cm laminated cards (each consisting of 20 small red dots in a 5 × 4 grid) attached at heights of 3 cm (essentially ground-level), 15 cm, 30 cm and 50 cm above the ground. For each height class, the number of red dots visible from a horizontal distance of 2 m was recorded.

At 2 m intervals along the transect, the maximum heights of the grass, forbs and small trees were recorded. The presence or absence of certain habitat components in contact with the base of the pole (such as bare ground, dung and rocks) was also recorded. The percentage canopy cover was recorded every 8 m along the transect by looking through a 4.5 cm diameter cylinder and estimating the percentage of vegetation within the cylinder. Distance to the closest tree was estimated every 4 m (Gibb & Parr, 2010). The surveys of both habitat structure and complexity were conducted in summer (February) and in winter (July) to determine seasonal variation.

### Ant assemblage diversity & composition: pitfall trapping

Pitfall trapping was carried out once during the summer (February 2009) when ants are most active (Parr et al., 2004). At each site, 20 pitfall traps were set up in a grid (5 × 4) with 10 m spacing between the traps. Each trap was 120 mm deep with a diameter of 52 mm and was filled with approximately 10 ml of 50/50 water/propylene glycol solution as a preservative (Parr & Chown, 2001; Parr et al., 2004) and a drop of detergent to reduce surface tension (Netshilaphala, Milton & Robertson, 2005). Traps were open for 5 days to catch a representative proportion of the ant species occurring in that area (Parr & Chown, 2001). After the traps were collected, the ants were separated from other invertebrates caught and stored in 70% ethanol. Ant specimens were identified to species or morphospecies level in the laboratory. A reference collection is deposited at the University of Pretoria.

### Ant foraging behaviour: experimental habitat treatments

For the natural habitat experiment, open bait cards were used in complex (bunch grass sites) and simple (grazing lawn sites) habitats. If there was a difference between the complex and simple habitats, a manipulated experiment was conducted to determine if this difference was due to habitat complexity or the composition of ants. The experimental design was similar to that used by Sarty, Abbott & Lester (2006) and Gibb & Parr (2010). The manipulated treatments were set up in both the complex and simple habitats in all 12 sites. At each site, the role of microhabitat complexity was tested using two experimental treatments: ‘fine’ and ‘coarse’ and two control treatments: ‘control’ and ‘bait card’ (i.e., a total of four treatments). There were six replicates of the four treatments (arena) in each site. Experimental treatment arenas were constructed using round transparent plastic containers (14.7 cm in diameter, 12.5 cm in height), containing a central observation chamber (see Sarty, Abbott & Lester, 2006, Fig. S1).

Containers for the fine and coarse treatments were filled with materials occurring in the surrounding area to create either complex or simple structural complexity. Materials selected consisted of grass and litter in the bunch grass experiments, and stones, grass and litter in the grazing lawn experiments. A gap analysis was conducted for both the bunch grass habitat and the grazing lawn habitat in both the summer and winter seasons. A grid of 18 regularly placed dots was placed over the fine and coarse manipulated experiments with a light source from the bottom. The gap closest to each dot was traced and measured to the nearest mm. In the grazing lawn, sizes for the complex treatment varied between 1.156 ± 0.936 (summer) and 0.639 ± 0.449 (winter), while sizes for the fine treatment varied between 3.548 ± 1.994 (summer) and 2.784 ± 1.850 (winter). In the bunch grass habitat, gap sizes ranged between 0.549 ± 0.766 (summer) and 0.341 ± 0.731 (winter) in the complex treatment and between 2.948 ± 1.333 (summer) and 1.844 ± 1.150 (winter) in the fine treatments.

Cotton wool was packed on top of these materials to prevent ants walking over the top of the experimental habitat. Ants were able to access the central chamber through 1 cm diameter holes, cut 1 cm apart, at the base of the observation chambers and the microhabitat chambers. Control treatments consisted of containers with no added structure, while bait card treatments consisted simply of a card on which the bait was placed. To attract a variety of ants, fish-based protein and honey (carbohydrate) baits were placed in the central chamber or on the bait card. The protein was cut into blocks (1*cm* × 1.5*cm*) and the honey was placed in a small vial lid (2.5 cm in diameter). A Petri dish lid was also placed over the central observation chamber to prevent the ants climbing in or out to ensure they travelled through the manipulated vegetation interstices.

Within each site, six arenas were placed in a randomised 4 m^2^ block design. Arenas were placed roughly 20 m apart. The experiment was started at approximately 08h00 and finished 3 h later. The temperature at the start of the experiment and every hour afterwards was recorded to account for ant activity during changes in temperature (Kaspari, 1993). To explore the effect of seasonal variation, this experiment was conducted in winter (June) and summer (December) 2008. Air temperatures while sampling in winter ranged from 24.1 °C–32.9 °C and 23.9 °C–31.3 °C for grazing lawn and bunch grass sites respectively. Summer temperatures were higher than winter temperatures: 28.6 °C–36.2 °C in bunch grass sites and 25.6 °C–37.5 °C in grazing lawn areas. After placement in the field, all replicates were observed continuously until they had been discovered. The identity of the first ant to discover the bait and the time taken until discovery were recorded. Where positive field identification was not possible, the ant was collected through the top of the observation chamber using forceps or an aspirator. Following bait discovery, observations were taken at hourly intervals for 3 h and the temperature, the identity of any ant species present and their abundance were recorded. Species that monopolised a resource after 3 h were considered to be competitively dominant at that resource, even if interference competition was not observed (“Numerical dominance” at baits, Parr et al., 2005; Gibb & Parr, 2010). Dominant species are considered to be those that monopolised the baits (i.e., were the only species present at the baits) and had an abundance of five or more workers. In the laboratory, six specimens of each species that arrived first at the baits were measured. For each ant, standard linear measurements were taken using an ocular micrometer mounted on a dissecting microscope accurate to 0.01 mm. These measurements were of the head width across the top of the eyes and the hind femur length. The Body Size Index (BSI) (see Sarty, Abbott & Lester, 2006; Gibb & Parr, 2010) was used as an overall indicator of body size. It was calculated as BSI = head width × hind femur length.

### Data analysis

To quantify the effect of grazing on vegetation complexity and structure, a two-way ANOVA using habitat and season was performed using SPSS version 15.0. Ant species richness and abundance were calculated for each site respectively in the grazing lawn and bunch grass habitat. The results were then used in a two-way ANOVA using habitat and species richness or abundance. To determine if grazing had a significant effect on ant assemblage composition, pitfall trap data were analysed using PRIMER version 6. Raw abundance data were fourth root transformed to weight appropriately rare and abundant species (Gibb & Hochuli, 2002). Analysis of Similarity (ANOSIM) (Chapman & Underwood, 1999) was used to test for significant differences in assemblage composition between bunch grass and grazing lawn habitats, and the data were ordinated using non-metric multidimensional scaling (nMDS). To establish how much each species contributed to the differences between habitats a SIMPER routine was conducted (Chan et al., 2008).

A three factor ANOVA model was used to analyse the effects of season and habitat structural complexity (manipulated and natural treatments) on the speed of resource discovery, the number of times baits were monopolised and ant species body size for both the natural and manipulated experiments. We predicted that during winter ants would locate resources at a slower rate and monopolise fewer resources. Where ANOVA results proved significant, a Kruskal–Wallis test was conducted to distinguish the order in which the species of ants arrived at resources, the frequency at which they monopolised the baits, or to determine the size class of the species. Tukey’s HSD *post-hoc* tests were conducted to determine which treatments were significantly different. A three-way MANOVA was conducted to determine if the dependent variables (monopolisation and discovery of baits at 60, 120 and 180 min) were significantly affected by differences in the independent variables (habitat and season). As each time interval is not independent, repeated ANOVA measures were used to analyse if there were significant differences between habitats and seasons over a period of 60, 120 and 180 min for ants discovering and monopolising resources.

## Results

### Vegetation structural complexity and composition

The two habitats differed significantly in structure and composition, with lower structural complexity (number of visible dots), reduced vegetation height and litter presence in the grazing lawn habitat for both seasons (Table 1). Importantly there was more bare ground in the grazing lawn habitat in summer and much more grass in the bunch grass habitat for both seasons (Table 1). Our results suggest a more complex environment in the bunch grass habitat compared with the grazing lawn habitat (Table 1).

**Table 1:** Measures of natural habitat complexity at ground level including the number of visible dots for vegetation complexity on the dot card (at various heights above ground level), vegetation height, and cover of bare ground, litter or dung cover, tree density.

**Habitat**	Summer				Winter			
	Bunch Grass	Grazing Lawn	*F*_(1,10)_	*P*-value	Bunch Grass	Grazing Lawn	*F*_(1,10)_	*P*-value
	(Complex)	(Simple)			(Complex)	(Simple)		
*Mean number of visible dots*								
3 cm	1.5	8.9	13	**0.0050**	0.87	11	76	**0.0**
15 cm	7.8	19	23	**0.0010**	5.2	19	235	**0.0**
30 cm	16	19	5.5	**0.041**	13	20	14	**0.0040**
50 cm	20	20	1.4	0.27	19	20	1.6	0.24
*Mean height (m)*								
Forb	0.030	0.013	0.54	0.48	0.013	0.012	0.024	0.88
Small woody	0.037	0.0090	4.1	0.070	0.049	0.0040	7.2	**0.023**
Grass	0.14	0.013	52	**0.000**	0.18	0.015	41	**0.0**
*Mean number of points*								
Dung	–	–	–	–	1.5	1.5	0.0	1.0
Litter	**–**	0.33	1.0	0.34	11	2.2	5.3	**0.045**
Bare Ground	9.5	19	9.7	**0.011**	25	29	0.64	0.44
Distance to closest tree (m)	3.2	2.5	0.17	0.69	2.8	4.0	0.74	0.41
% canopy cover	11	9.2	0.21	0.66	7.4	3.1	1.1	0.33

**Notes.**

Bold indicates significant values.

### Ant assemblage diversity & composition

**Figure 2:**
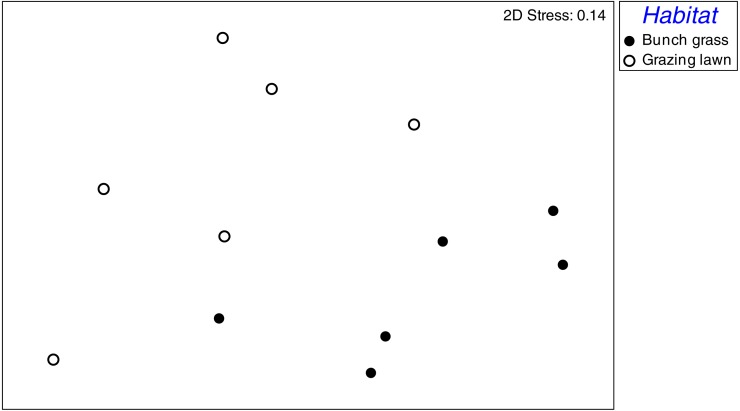
Non-metric multidimensional scaling ordination for bunch grass and grazing lawn ant assemblages. ANOSIM: Global *R* = 0.4 and *P* = 0.0004.

A total of 57 species was recorded using both sampling methods, with 40 species from the grazing lawn sites and 48 species from the bunch grass sites. Pitfall trap data showed there were no significant differences in either abundance or species richness between habitats (ANOVA, *F*_(1,10)_ = 2.0, *P* = 1.8 and *F*_(1,10)_ = 0.39, *P* = 0.55 for abundance and richness respectively). However, ant assemblage composition differed significantly between the grazing lawn and the bunch grass habitats (Global *R* = 0.41, *P* = 0.006) (Fig. 2). Four species contributed more than 23% of the dissimilarity between the two habitats: *Anoplolepis custodiens* (12%), *Myrmicaria natalensis* (5.1%), *Pheidole* sp. A (4.6%), and *Pheidole* sp. H (4.0%) (Table 2).

**Table 2:** A one-way SIMPER routine indicating the dissimilarity of ant species between the bunch grass habitat and grazing lawn habitat from the pitfall traps.

Ant species	Average abundance in the bunch grass habitat	Average abundance in the grazing lawn habitat	% contribution to dissimilarity
*Anoplolepis custodiens*	1.2	5.6	12
*Myrmicaria natalensis*	2.3	0	5.1
*Pheidole* Sp. A	3.2	3	4.6
*Pheidole* Sp. H	1.7	1.6	4.0
*Tetramorium sericeiventre*	2.0	3.3	3.8

### Resource discovery

#### Natural habitats

**Figure 3:**
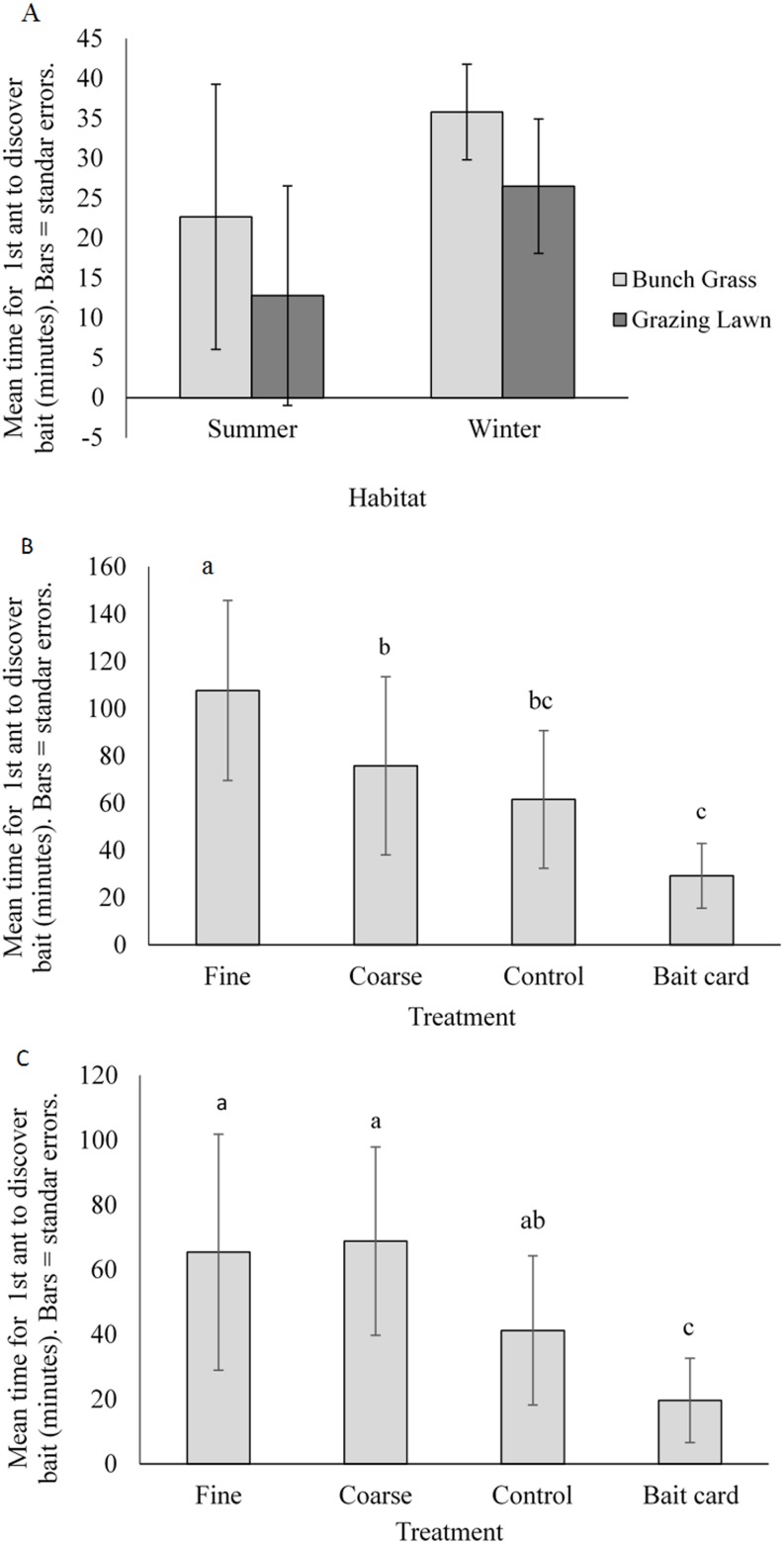
Mean (±SE) time for first ant to discover baits for (A) natural habitats by season, and for manipulated treatments: (B) complex bunch grass habitat and (C) simple grazing lawn habitat. Bars with different letters are significantly different.

In the natural experiment (data for summer and winter combined), there was no significant difference in the rate of resource discovery between habitats (ANOVA, *F*_(1,20)_ = 3.9, *P* = 0.063, mean time for first ant to discover a bait ± SE = 20 ± 13 and 29 ± 14 for grazing lawn and bunch grass habitats respectively). There was, however, a seasonal effect, with ants taking longer to arrive at the baits in winter than in summer (*F*_(1,20)_ = 7.6, *P* = 0.0012, see also Fig. 3A).

There was no significant difference in resource discovery between habitats and season for all three time periods; 60, 120 and 180 min (Table 3). Seasonally there was a significant difference at 60 min with ants discovering baits faster in summer than winter (MANOVA, *F*_(1,20)_ = 8.9, *P* = 0.007) (Table 3). Repeated ANOVA measures confirm that there was no significant difference in ants discovering baits between habitats over time (*F*_(2,40)_ = 1.1, *P* = 0.35). It also confirmed that there was a significant difference between the seasons (ANOVA, *F*_(2,40)_ = 8.1, *P* = 0.001) with ants discovering baits faster in summer (Table 3).

When looking at habitat and season combined, repeated ANOVA measures indicated that the number of baits discovered by a single ant or more increased significantly with time (ANOVA, _(2,40)_ = 9.1, *P* = 0.001).

#### Experimental habitat treatments

In both the grazing lawn habitat and bunch grass habitat, there were significant differences between the experimental treatments in the speed of resource discovery (ANOVA, grazing lawn habitat, *F*_(3,40)_ = 9.7, *P* < 0.001; Bunch grass habitat, *F*_(3,40)_= 15, *P* < 0.001) (Table 3, Fig. 3). Kruskal–Wallis tests revealed that the open bait card treatments were discovered first followed by the control in both the bunch grass (Fig. 3B) and grazing lawn habitats (Fig. 3C). Tukey’s *post-hoc* tests revealed that the bait card treatment was discovered significantly faster than the control, coarse and fine treatments in the bunch grass habitat (Fig. 3B). In the grazing lawn habitat, the bait card treatment was discovered at a significantly faster rate than the coarse and fine treatments (Fig. 3C).

**Table 3:** Mean number of baits discovered and monopolised in the bunch grass habitat and the grazing lawn habitat for the summer and winter seasons over the three time periods (60, 120 and 180 min).

	**Bunch Grass**		**Grazing Lawn**		**Two-way MANOVA**			
	Summer	Winter	Summer	Winter	Habitat		Season	
					*F*_(1,20)_	*P*-value	*F*_(1,20)_	*P*-value
*Mean number of baits discovered ^a^*								
60 mins	5.8 (.41)	4.8 (.75)	5.8 (.41)	5.5 (.55)	2.2	0.15	8.9	**0.007**
120 mins	5.5 (.84)	5.8 (.41)	5.8 (.41)	6.0 (.00)	1.5	0.24	1.45	0.24
180 mins	6.0 (.00)	6.0 (.00)	6.0 (.00)	6.0 (.00)	–	–	–	–
*Mean number of baits monopolised ^b^*								
60 mins	2.0 (.90)	4.3 (1.0)	4.8 (.75)	4.0 (.1.7)	7.2	**0.014**	2.6	0.12
120 mins	3.0 (1.1)	4.7 (1.5)	4.3 (1.5)	4.3 (1.0)	0.88	0.36	2.5	0.13
180 mins	3.2 (1.3)	5.2 (1.2)	3.3 (2.4)	4.3 (1.4)	0.25	0.63	5.0	**0.037**

**Notes.**

a Repeated measure ANOVA between habitats, *F* = 1.1, *p* = 0.35.

b Repeated measure ANAOVA between habitats, *F* = 3.5, *p* = 0.040.

Bold indicates significant values.

### Resource monopolisation

#### Natural habitats

**Figure 4:**
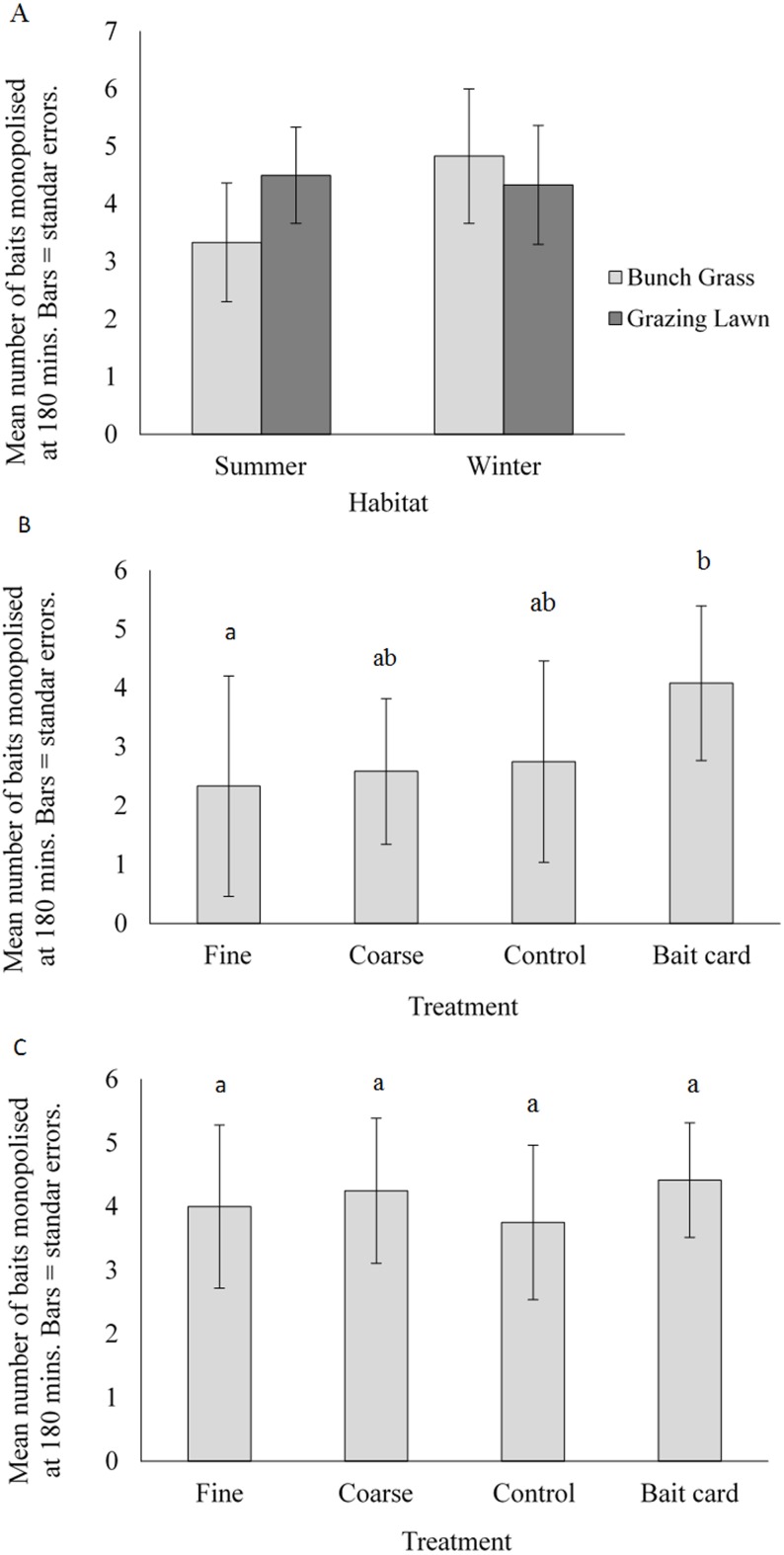
Mean (±SE) number of baits monopolised at 180 minutes: (A) natural habitat for summer and winter periods, and manipulated treatments in (B) the bunch grass habitat and (C) grazing lawn habitat. Bars with different letters are significantly different.

For seasons combined, there was no significant difference of resource monopolisation between the grazing lawn and bunch grass habitat (ANOVA, *F*_(1,20)_ = 0.64, *P* =  < 0.44, mean number of baits monopolised = 4.4 ± SE 0.90 and 4.1 ± 1.3 for the grazing lawn and bunch grass habitats respectively) (Table 3, Fig. 4). When habitats were combined, season had no significant impact on the number of monopolised baits (ANOVA, *F*_(1,80)_ = 2.5, *P* = 0.13).

At 60 min there was a significant difference between habitats (Table 3), with more ants monopolising baits in the grazing lawn habitat compared with the bunch grass habitat (MANOVA, *F*_(1,20)_ = 7.2, *P* = 0.014). Seasonally there was a significant difference only at 180 min (MANOVA, *F*_(1,20)_ = 5.0, *P* = 0.037) when there were more baits monopolised in winter than in summer (Table 3). There was a significant difference in monopolisation of baits with the interaction between habitat and time (ANOVA, *F*_(2,40)_ = 3.5, *P* = 0.040), with ants monopolising more baits in the grazing lawn sites early on in the experiment (Table 3). Repeated ANOVA measures confirm that when looking at the interaction between season and time, there was no significant difference (ANOVA, *F*_(2,40)_ = 0.94, *P* = 0.40). When season and habitat were combined, repeated ANOVA measures indicated that there was no significant difference in bait monopolisation over time (ANOVA, *F*_(2,40)_ = 0.50, *P* = 0.61).

#### Manipulated experiment

Habitat treatment affected the number of baits monopolised (summer and winter data combined) in the bunch grass habitat (ANOVA, *F*_(3,40)_ = 3.6, *P* = 0.021), but not the grazing lawn habitat (ANOVA, *F*_(3,40)_ = 0.76, *P* = 0.52) (Figs. 4B & 4C). Tukey’s *post-hoc* tests indicated that in the bunch grass habitat the bait card treatment had significantly higher levels of monopolisation than the fine and coarse treatments (Fig. 4B). There was, however, no significant difference between any of the other treatments (Fig. 4B).

### The effect of habitat structure on Body Size Index

**Figure 5:**
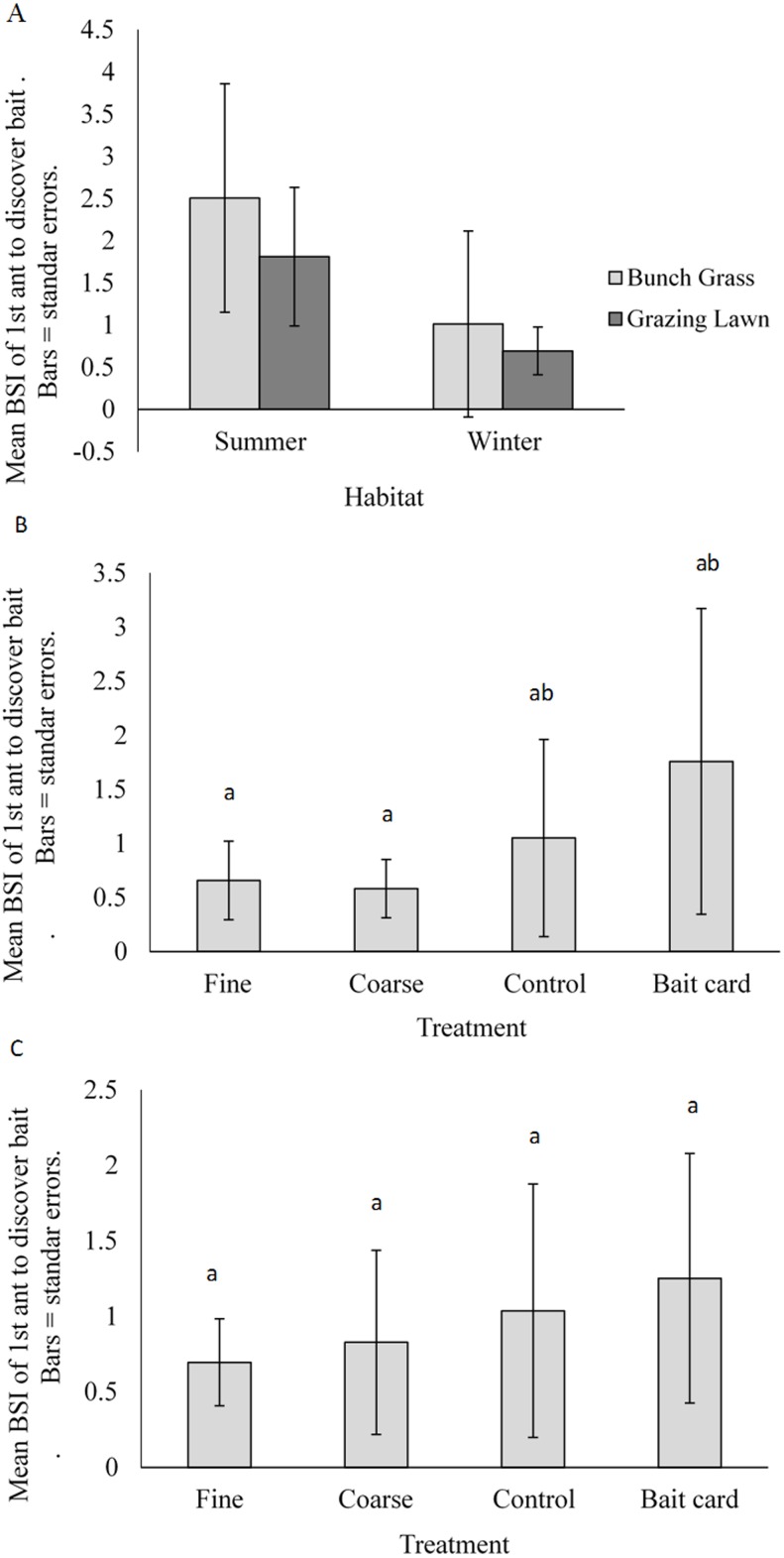
Mean (±SE) Body Size Index (BSI) of ants for (A) natural habitats (summer and winter seasons), and manipulated treatments in (B) the bunch grass habitat and (C) grazing lawn habitat. Bars with different letters are significantly different.

A three-factor full model ANOVA yielded a non-significant result (*F*_(1,80)_ = 1.6, *P* = 0.22) for the interaction between BSI and habitat (mean body size ± SE: 1.8 ± 1.4 and 1.3 ± 0.83 for the bunch grass and grazing lawn habitats respectively). Overall, ant species active in the summer had larger mean BSI’s (2.16 ± 1.1), whereas the species of ants recorded in winter were significantly smaller (0.850 ± 0.79; ANOVA, *F*_(1,20)_ = 11, *P* = 0.0040) (Fig. 5A).

In accordance with predictions, BSI differed significantly with the manipulated treatments in the bunch grass habitat (seasons combined) (ANOVA, *F*_(1,40)_ = 5.3, *P* = 0.004), but not in the grazing lawn habitat (ANOVA, *F*_(1,40)_ = 2.0, *P* = 0.13) (Fig. 5B & 5C). Tukey’s *post-hoc* tests revealed that the bait card treatment (BSI = 1.8 ± 1.4) had a significantly higher BSI than the coarse (0.58 ± 0.27) and fine (0.66 ± 0.36) treatments. The control (1.1 ± 0.91) treatment, however, remained similar to the bait, coarse and fine treatments in the bunch grass habitat (Fig. 5B).

## Discussion

As with other taxa (e.g., plants, birds and spiders; Krook, Bond & Hockey, 2007; Hempson et al., 2015), intense grazing by large mammalian herbivores has a significant effect on local ant diversity and functioning. Our study shows that the creation of grazing lawns by large mammals plays an important role in promoting local heterogeneity as ant assemblage composition differed significantly between the two habitats. By combining natural and manipulated experiments, a deeper insight was gained into the foraging behaviour of ants in response to changing habitat structural complexity. Our results for the natural experiments did not support our predictions as there was no difference in the rate of resource discovery between the two habitats. In part, our predictions for bait monopolisation were supported as more ants dominated baits in the simple grazing lawn habitat, but only within the first 60 min of the experiment. In the manipulated experiments, simple bait treatments were located faster, but there was a difference in monopolisation only within the bunch grass habitat. The hypothesis that ant body size changes as a function of habitat complexity was not supported in the natural experiment. The manipulated experiment from the bunch grass habitat did support our hypothesis as the more complex bait treatments attracted ants with smaller BSI. Seasonally, predictions aligned in part with our hypothesis, as ants took longer to locate resources in winter but monopolised them at a higher rate. Our study demonstrated that changes in habitat structure as a result of grazing have significant implications for the way these systems function through changes to ant foraging behaviour and consequently to foraging success.

### Natural experiment

This study shows that grazing causes the simplification of the ground layer structure, which was predicted to influence ant behaviour as well as their assemblages. In structurally simple habitats, other studies have shown that ants are better able to locate and capture food resources than in complex habitats (Pik et al., 2002; Lassau & Hochuli, 2004; Gibb & Parr, 2010). Our data, however, do not support this prediction, as we found no difference in the abundance of ants between habitats.

A plausible explanation for this is that it takes ants longer to move through a structurally complex habitat and thus locate and capture food resources. The complex ground layer of the vegetation requires ants to climb through, round and over the vegetation rather than traverse the surface. Energetic costs of movement are increased in more complex environments reducing ant abundance and retrieval of resources (Lassau & Hochuli, 2004; Sarty, Abbott & Lester, 2006; Nakamura, 2008; Gibb & Parr, 2010). Chemical cues left by other ants are also harder to follow and locate, increasing the amount of time it takes to discover resources (Lassau & Hochuli, 2004; Lassau et al., 2005). For example, Parr et al. (2007) demonstrated that the simplification of savanna ground-layer habitat, due to disturbances such as fire, enables ants to remove greater quantities of seeds and transfer them over longer distances.

This study demonstrated that on a habitat scale, habitat complexity did not alter the rate of resource discovery. It did, however, alter the monopolisation of baits, but only within the first 60 min of the experiment. This suggests that ants find it easier to dominate food resources in simple habitats but that there are also other factors that influence ant behaviour at a habitat scale. For example, season and consequently temperature could alter their behaviour.

### Manipulated experiment

Results from the manipulated experiment support the finding that ants locate resources faster in the more simple treatments (bait cards and control). However, the different treatments demonstrated no difference in the numbers of baits monopolised. This could be related to scale because the natural experiments as mentioned above indicated that simple habitats supported higher monopolisation of ants at resources in the first hour of the experiment. Overall, dominance is promoted in more simple environments. Our data from the bunch grass site also suggest that within a given habitat there is a filtering effect of habitat complexity.

Findings from the manipulated experiment support the size-grain hypothesis (Kaspari & Weiser, 1999) but not in the natural experiment as BSI did not vary. The size grain hypothesis appears to be affected by the scale of the study. Only at a micro-habitat scale (in the manipulated treatments) does it appear to be important. Habitat structural complexity acts as a filter for body size, therefore restricting certain species of a particular size class (Kaspari & Weiser, 1999; Parr, Parr & Chown, 2003; Farji-Brener, 2004; Sarty, Abbott & Lester, 2006; Nakamura, 2008; Gibb & Parr, 2010).

### Season

We predicted that season would influence the time it takes ants to locate and monopolise resources. This is because in winter ant activity is generally reduced. Therefore, the chance of finding and monopolising baits is also reduced (McGrannachan & Lester, 2013). Temperature variations have been shown to alter foraging patterns and competitive interactions of ants, resulting in species occupying different niches (Cerda, Retana & Cros, 1998; Barbieri, Grangier & Lester, 2015). Habitat complexity has also been shown to affect temperatures because in structurally simple habitats radiation penetration is higher than in structurally complex habitats. This could affect ant behaviour and consequently alter the time of day in which ants are more active (Lassau & Hochuli, 2004; Lassau et al., 2005).

Results from this study indicate that ants take longer to locate resources in winter but only within the first 60 min. After the first hour of the experiment air temperature was probably warm enough not to affect ant behaviour in both habitats, and by 120 min it may have been too hot.

Even though ants can exist in areas with higher temperatures, because of their small body size they are prone to desiccation (Kaspari, 1993). As it was not logistically possible to start the experiment before 08h00, temperatures at the end of the experiment (especially in summer) were often extremely high (air temperature > 30 °C), and consequently activity at baits declined between the 120 and 180 min periods (Table 3). High ground surface temperatures prevented foraging by all but the most thermophilic species. This may explain the presence of larger ants (*Anoplolepis custodiens*, *Odontomachus* spp.*, Myrmicaria natalensis* and *Ocymyrmex fortior*) during the summer season and the lower levels of bait monopolisation at the simple grazing lawn habitat after 180 min within the natural experiments.

## Conclusions

This study has demonstrated that grazing by large mammalian herbivores can indirectly influence not only assemblage structure but also foraging success and behaviour of ants through changes to habitat structure. This shows that habitat structure is important to consider in conservation biology as ants have been shown to play a pivotal role in ecosystem engineering and functioning (Folgarait, 1998; Parr et al., 2016; Griffiths et al., 2018). Parr et al. (2016) demonstrated that ants initiate top-down control of termites and can structure the invertebrate community more widely. Nutrient hotspots (e.g., termite mounds) and disturbances such as grazing which facilitate the formation of grazing lawns are important for maintaining heterogeneity within the savanna biome (Krook, Bond & Hockey, 2007; Davies et al., 2014). This heterogeneity enables species co-existence thus promoting diversity. Although disturbances can create heterogeneity within a habitat, they can also result in the homogenising of habitat, for example through the introduction of invasive plant species (Crooks, 2002). The presence of a mix of grazing lawns and bunch grass habitats and their associated faunal assemblages in savannas is essential, and it is therefore imperative that efforts should focus on conserving species considered critical for the maintenance of grazing lawns (e.g., white rhino).

##  Supplemental Information

10.7717/peerj.6226/supp-1 Supplemental Information 1 Raw Data for the discovery, dominance and monopolisation of baits by ants in the manipulated and natural experiments. It also includes data on ant assemblages from the pitfall traps and the sizes of ants found in both the experimental and natural experime

10.7717/peerj.6226/supp-2 Figure S1 A Visual representation of the manipulated experiments

## Additional Information and Declarations

### Field Study Permissions

The following information was supplied relating to field study approvals (i.e., approving body and any reference numbers): Field permits were approved by KZN Ezemvelo Wildlife. Field permit number is 1858/2008.

### Data Availability

The following information was supplied regarding data availability: The raw data are available as Supplementary Files. It includes the vegetation complexity data, ant monopolisation, dominance and discovery data for the manipulated and experimental treatments, data from the pitfall traps, determining biodiversity of grazing lawns and bunch grass habitats and ant size measurements of the first ant to reach the manipulated experiments.

## References

[ref-1] Almany GR (2004). Differential effects of habitat complexity, predators and competitors on abundance of juvenile and adult coral reef fishes. Oecologia.

[ref-2] Andersen AN (1991). Responses of ground-foraging ant communities to three experimental fire regimes in a savanna forest of tropical Australia. Biotropica.

[ref-3] Andersen AN (1995). A classification of Australian ant communities, based on functional groups which parallel plant life-forms in relation to stress and disturbance. Journal of Biogeography.

[ref-4] Archibald S, Bond WJ, Stock WD, Fairbanks DHK (2005). Shaping the landscape: fire-grazer interactions in an African savanna. Ecological Applications.

[ref-5] Barbieri RF, Grangier J, Lester PJ (2015). Synergistic effects of temperature, diet and colony size on the competitive ability of two ant species. Austral Ecology.

[ref-6] Cerda X, Retana J, Cros S (1998). Critical thermal limits in Mediterranean ant species: trade-off between mortality risk and foraging performance. Functional Ecology.

[ref-7] Chan EKW, Yu YT, Zhang Y, Dudgeon D (2008). Distribution patterns of birds and insect prey in a tropical riparian forest. Biotropica.

[ref-8] Chapman M, Underwood A (1999). Ecological patterns in multivariate assemblages: information and interpretation of negative values in ANOSIM tests. Marine Ecology Progress Series.

[ref-9] Coughenour MB (1985). Graminoid responses to grazing by large herbivores: adaptations, exaptations and interacting processes. Annals of the Missouri Botanical Garden.

[ref-10] Cromsigt JPGM (2006). Large herbivores in space: resource partitioning among savanna grazers in a heterogenous environment. D. Phil. Thesis.

[ref-11] Cromsigt JPGM, Veldhuis M, Stock W, Roux E le, Gosling C, Archibald S, Cromsigt JPGM, Archibald S, Owen-Smith N (2017). The functional ecology of grazing lawns—how grazers, termites, people, and fire shape HiP’s savanna grassland mosaic. Conserving Africa’s mega-diversity in the Anthropocene. The Hluhluwe-iMfolozi Park story.

[ref-12] Crooks JA (2002). Characterizing ecosystem-level consequences of biological invasions: the role of ecosystem engineers. Oikos.

[ref-13] Davies AB, Levick SR, Asner GP, Robertson MP, Rensburg BJ van, Parr CL (2014). Spatial variability and abiotic determinants of termite mounds throughout a savanna catchment. Ecography.

[ref-14] Farji-Brener AG (2004). Comment—the size-grain hypothesis in ants: conflicting evidence or confounded perspective?. Ecological Entomology.

[ref-15] Folgarait PJ (1998). Ant biodiversity and its relationship to ecosystem functioning: a review. Biodiversity & Conservation.

[ref-16] Gibb H, Hochuli DF (2002). Habitat fragmentation in an urban environment: large and small fragments support different arthropod assemblages. Biological Conservation.

[ref-17] Gibb H, Parr CL (2010). How does habitat complexity affect ant foraging success? A test using functional measures on three continents. Oecologia.

[ref-18] Grabowski JH (2012). Habitat complexity disrupts predator prey interactions but not the trophic cascade on oyster reefs. Ecology.

[ref-19] Griffiths HM, Ashton LA, Walker AE, Hasan F, Evans TA, Eggleton P, Parr CL (2018). Ants are the major agents of resource removal from tropical rainforests. Journal of Animal Ecology.

[ref-20] Hempson GP, Archibald S, Bond WJ, Ellis RP, Grant CC, Kruger FJ, Kruger LM, Moxley C, Owen-Smith N, Peel MJS, Smit IPJ, Vickers KJ (2015). Ecology of grazing lawns in Africa. Biological Reviews.

[ref-21] Howison RA, Olff H, Van de Koppel J, Smit C (2017a). Biotically driven vegetation mosaics in grazing ecosystems: the battle between bioturbation and biocompaction. Ecological Monographs.

[ref-22] Howison RA, Olff H, Owen-Smith N, Cromsigt JPGM, Archibald S, Cromsigt JPGM, Archibald S, Owen-Smith N (2017b). The abiotic template for the Hluhluwe-iMfolozi Park’s landscape heterogeneity. Conserving Africa’s mega-dversity in the Anthropocene. The Hluhluwe-iMfolozi Park story.

[ref-23] Hurlbert AH (2004). Species-energy relationships and habitat complexity in bird communities. Ecology Letters.

[ref-24] James CD (2004). Trapping intensities for sampling ants in Australian rangelands. Austral Ecology.

[ref-25] Kaspari M (1993). Body size and microclimate use in neotropical granivorous ants. Oecologia.

[ref-26] Kaspari M, Weiser MD (1999). The size-grain hypothesis and interspecific scaling in ants. Functional Ecology.

[ref-27] Krook K, Bond WJ, Hockey PAR (2007). The effect of grassland shifts on the avifauna of a South African savanna. Ostrich.

[ref-28] Lassau S, Hochuli D (2004). Effects of habitat complexity on ant assemblages. Ecography.

[ref-29] Lassau SA, Hochuli DF (2005). Wasp community responses to habitat complexity in Sydney sandstone forests. Austral Ecology.

[ref-30] Lassau SA, Hochuli DF, Cassis G, Reid CAM (2005). Effects of habitat complexity on forest beetle diversity: do functional groups respond consistently?. Diversity & Distributions.

[ref-31] Macdonald IAW (1983). Alien trees, shrubs and creepers invading indigenous vegetation in the Hluhluwe-Umfolozi Game Reserve complex in Natal. Bothalia.

[ref-32] McGrannachan CM, Lester PJ (2013). Temperature and starvation effects on food exploitation by Argentine ants and native ants in New Zealand. Journal of Applied Entomology.

[ref-33] McNaughton SJ (1979). Grazing as an optimization process: grass-ungulate relationships in the Serengeti. American Naturalist.

[ref-34] McNaughton SJ (1985). Ecology of a grazing ecosystem: the Serengeti. Ecological Monographs.

[ref-35] Nakamura N (2008). Effect of habitat complexity on ant community organisation. D. Phil. Thesis.

[ref-36] Netshilaphala NM, Milton SJ, Robertson HG (2005). Response of an ant assemblage to mining on the arid Namaqualand coast, South Africa: short communication. African Entomology.

[ref-37] Oliver TH, Heard MS, Isaac NJB, Roy DB, Procter D, Eigenbrod F, Freckleton R, Hector A, Orme CDL, Petchey OL, Proença V, Raffaelli D, Suttle KB, Mace GM, Martín-López B, Woodcock BA, Bullock JM (2015). Biodiversity and resilience of ecosystem functions. Trends in Ecology and Evolution.

[ref-38] Painter EL, Belsky AJ (1993). Application of herbivore optimization theory to rangelands of the western United States. Ecological Applications.

[ref-39] Parr CL, Andersen AN, Chastagnol C, Duffaud C (2007). Savanna fires increase rates and distances of seed dispersal by ants. Oecologia.

[ref-40] Parr CL, Chown SL (2001). Inventory and bioindicator sampling: testing pitfall and Winkler methods with ants in a South African savanna. Journal of Insect Conservation.

[ref-41] Parr CL, Eggleton P, Davies AB, Evans TA, Holdsworth S (2016). Suppression of savanna ants alters invertebrate composition and influences key ecosystem processes. Ecology.

[ref-42] Parr CL, Robertson HG, Biggs HC, Chown SL (2004). Response of African savanna ants to long-term fire regimes. Journal of Applied Ecology.

[ref-43] Parr CL, Sinclair BJ, Andersen AN, Gaston KJ, Chown SL (2005). Constraint and competition in assemblages: a cross-continental and modeling approach for ants. The American Naturalist.

[ref-44] Parr ZJE, Parr CL, Chown SL (2003). The size-grain hypothesis: a phylogenetic and field test. Ecological Entomology.

[ref-45] Pik AJ, Dangerfield JM, Bramble RA, Angus C, Nipperess DA (2002). The use of invertebrates to detect small-scale habitat heterogeneity and its application to restoration practices. Environmental Monitoring and Assessment.

[ref-46] Sanders D, Platner C (2006). Intraguild interactions between spiders and ants and top-down control in a grassland food web. Oecologia.

[ref-47] Sarty M, Abbott KL, Lester PJ (2006). Habitat complexity facilitates coexistence in a tropical ant community. Oecologia.

[ref-48] Tews J, Brose U, Grimm V, Tielborger K, Wichmann MC, Schwager M, Jeltsch F (2004). Animal species diversity driven by habitat heterogeneity/diversity: the importance of keystone structures. Journal of Biogeography.

[ref-49] Waldram M (2005). The ecological effects of grazing by the white rhino (*Ceratotherium simum simum*) at a landscape scale. D. Phil. Thesis.

[ref-50] Waldram MS, Bond WJ, Stock WD (2008). Ecological engineering by a mega-grazer: white Rhino impacts on a South African savanna. Ecosystems.

[ref-51] Whateley A, Porter RN (1983). The woody vegetation communities of the Hluhluwe-Corridor- Umfolozi Game Reserve Complex. Bothalia.

