# Supplementary figures and images for: Grazing by large savanna herbivores indirectly alters ant diversity and promotes resource monopolisation
Source: PeerJ. 2019 Jan 11;7:e6226. doi: 10.7717/peerj.6226 (PMC6330944; doi:10.7717/peerj.6226)

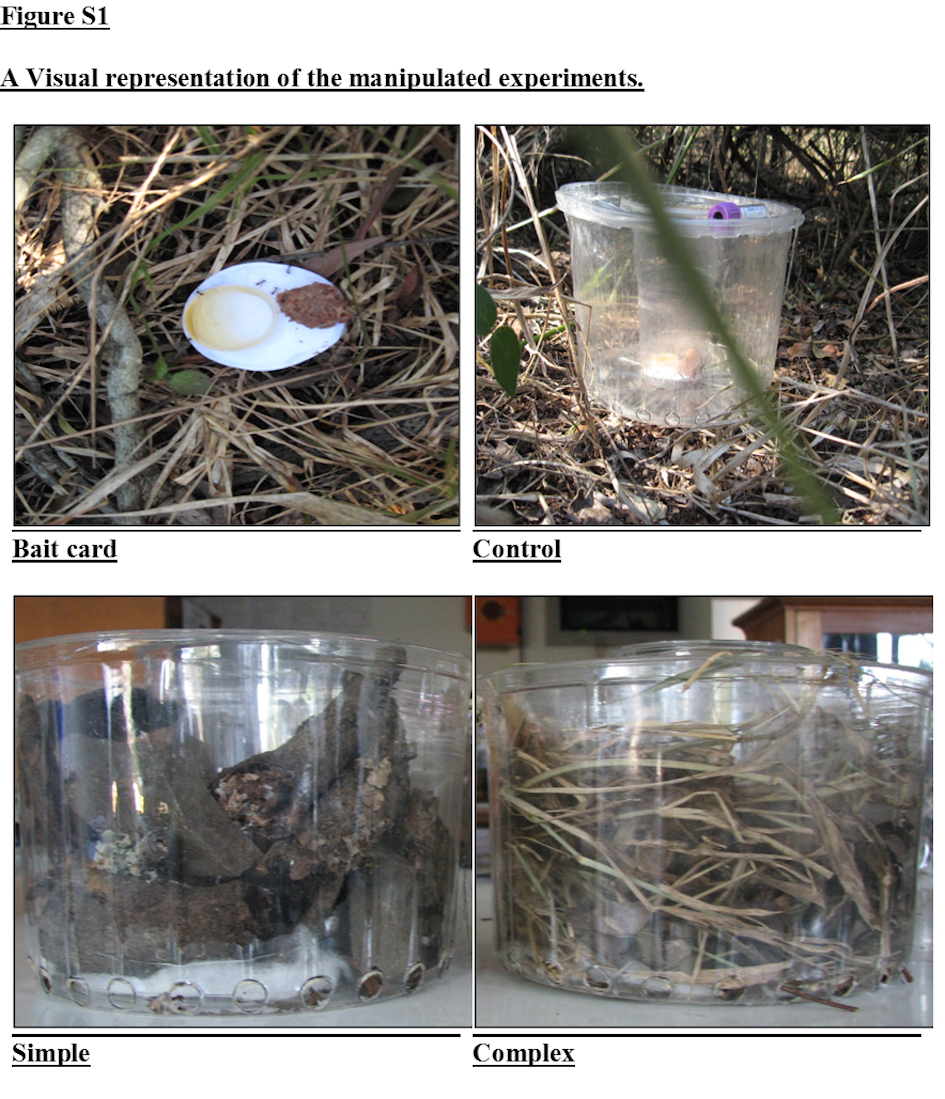

Supplement: Figure S1 [file peerj-07-6226-s002.png]
